# Maternal viral load monitoring: Coverage and clinical action at 4 Kenyan hospitals

**DOI:** 10.1371/journal.pone.0232358

**Published:** 2020-05-29

**Authors:** Matthew Sandbulte, Melinda Brown, Catherine Wexler, May Maloba, Brad Gautney, Kathy Goggin, Elizabeth Muchoki, Shadrack Babu, Nicodemus Maosa, Sarah Finocchario-Kessler

**Affiliations:** 1 Department of Family Medicine, University of Kansas Medical Center, Kansas City, KS, United States of America; 2 Global Health Innovations, Nairobi, Kenya; 3 Global Health Innovations, Dallas, TX, United States of America; 4 Health Services and Outcomes Research, Children’s Mercy Kansas City, Kansas City, MO, United States of America; 5 School of Medicine, University of Missouri-Kansas City, Kansas City, MO, United States of America; 6 Kenya Medical Research Institute, Nairobi, Kenya; University of North Carolina at Chapel Hill, UNITED STATES

## Abstract

**Background:**

Kenya’s guidelines for prevention of mother-to-child transmission of HIV (PMTCT) recommend routine viral load (VL) monitoring for pregnant and breastfeeding women.

**Method:**

We assessed PMTCT VL monitoring and clinical action occurring between last menstrual period (LMP) and 6 months postpartum at 4 Kenyan government hospitals. Pregnant women enrolled in the HIV Infant Tracking System from May 2016-March 2018 were included. We computed proportions who received VL testing within recommended timeframes and who received clinical action after unsuppressed VL result.

**Results:**

Of 424 participants, any VL testing was documented for 305 (72%) women and repeat VL testing was documented for 79 (19%). Only 115 women (27%) received a guideline-adherent baseline VL test and 27 (6%) received a guideline-adherent baseline and repeat VL test sequence. Return of baseline and repeat VL test results to the facility was high (average 96%), but patient notification of VL results was low (36% baseline and 49% repeat). Clinical action for unsuppressed VL results was even lower: 11 of 38 (29%) unsuppressed baseline results and 2 of 14 (14%) unsuppressed repeat results triggered clinical action.

**Discussion:**

Guideline-adherent VL testing and clinical intervention during PMTCT must be prioritized to improve maternal care and reduce the risk of HIV transmission to infants.

## Introduction

Prevention of mother-to-child transmission (PMTCT) services for pregnant women and mothers living with HIV throughout the antenatal, delivery, and postpartum phases are critical to reducing perinatal transmission and preserving the health of the mother and child. Despite significant uptake of antiretroviral therapy (ART) among pregnant women with HIV, adoption of recommended regimens has been gradual, with only 38% of pregnant women at selected Kenyan facilities receiving guideline adherent treatment between 2013 and 2016. [1] The goal of ART is to suppress maternal viral load (VL) to substantially lower risk of transmitting the virus to their infant during pregnancy [2–6] and breastfeeding. [7, 8] However, in several Sub-Saharan African PMTCT study populations, the proportions of pregnant women with unsuppressed VL range from 6.1–15.4%, [9–13] with 9.4–22% experiencing postpartum episodes of virologic rebound [14, 15]. This is consistent with the reported postpartum drop-off in Option B+ ART adherence. [16]

Routine maternal VL level provides a mechanism to identify elevated maternal viral load early so that corrective action can be taken to suppress maternal viral load (enhanced adherence counseling, drug resistance testing, regimen switch) and/or reduce the risk of transmission (intensified infant prophylaxis). [2, 17] As such, the World Health Organization recommended routine viral load testing in 2016 [18], with the ultimate objective of achieving maternal viral suppression before delivery and maintaining it through breastfeeding and beyond. Kenya adopted these guidelines in 2016. However, expanding VL testing for HIV-positive populations in low- and middle-income countries poses financial, logistical, and clinical operations challenges. [19–21] Results of studies from Senegal, Mozambique, and Kenya indicate that uptake of VL testing among pregnant women receiving ART are low: ranging from 36%-57%. [9, 10, 22] To the best of our knowledge, no studies to date have evaluated actual VL monitoring against the recommended guidelines to assess the timing of VL monitoring and compliance with repeat testing.

The objective of the present study was to evaluate implementation of Kenya’s PMTCT VL testing guidelines at four facilities, including documented baseline and repeat tests, return of test results, and clinical action taken in response to ART treatment failure, highlighting gaps and opportunities for strategic intervention.

## Methods

### Study setting

#### Kenyan viral load monitoring guidelines

**Fig 1:**
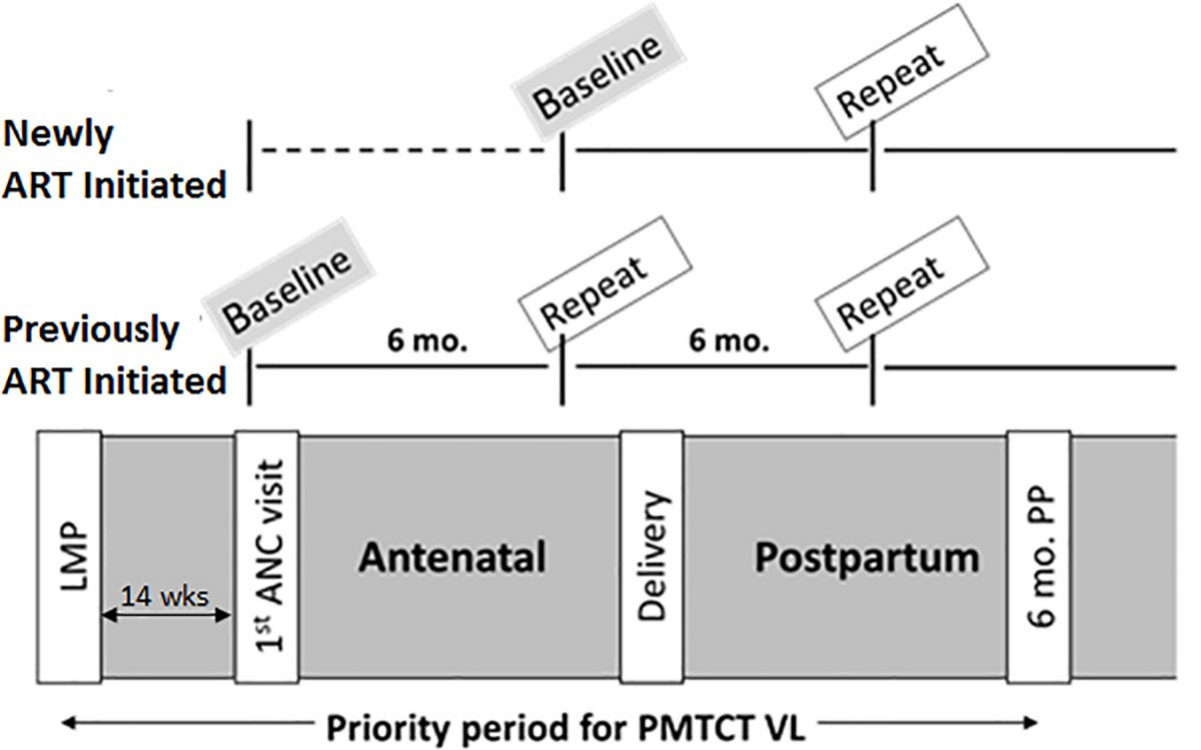
Optimal VL test sequences for HIV-positive pregnant women through 6 months postpartum. According to Kenya’s 2016 guidelines, pregnant women diagnosed with HIV in ANC are scheduled for baseline VL testing after 6 months on ART. Patients previously initiated on ART before conception are scheduled for baseline VL testing at first ANC visit. For virally suppressed patients in either category, the guidelines recommend repeat VL testing at 6 month intervals.

At the time of the study, Kenya’s PMTCT guidelines recommend early antenatal attendance, ART initiation <14 weeks gestation, and routine VL monitoring of pregnant and breastfeeding women. [23–25] Guideline-adherent VL monitoring schedules differ according to a patient’s PMTCT history: baseline testing for pregnant women previously diagnosed with HIV and initiated on ART is recommended at confirmation of pregnancy, while baseline testing for pregnant women newly initiated on ART is targeted to occur after 6 months of treatment (Fig 1). [26] Repeat testing for pregnant/breastfeeding women with suppressed VL is recommended at intervals of 6 months; repeat testing for those with unsuppressed VL is indicated within 3 months. [26] Fig 1 outlines PMTCT VL testing schedules per Kenya’s 2016 national guidelines, which were in effect during the time period targeted in this study.

#### Kenyan health care landscape

Kenya has over 9,000 health facilities, across six levels of care. Level 1–3 facilities (community health, dispensaries, health centers) focus on primary care and community health. Facility levels 4 and 5 (subcounty and county hospital, provincial hospital) provide more specialized services, including HIV care and maternity services, while level 6 facilities (national referral hospital) provide highly specialized services, in addition to HIV care and maternity services. Since 2013, maternity services (including ANC care, labor/delivery) have been provided free of charge in government facilities. [27] In the entirety of Kenya, there are approximately 16 nurses and 2 physicians per 10,000 population, however the geographic distribution of these providers varies substantially between counties. [27]

#### Study hospitals

We conducted a retrospective record review to evaluate guideline-adherent VL testing during PMTCT at four Kenyan government hospitals. The four government hospitals included in the study were in geographically diverse cities and ranged from level 4 to level 6, to capture the range of facilities that offer both HIV and maternity services. Details of the study hospitals are shown in Table 1.

**Table 1:** Facility details.

	Location	Facility Level^a^	Monthly PMTCT enrollment	Bed Capacity^b^	Clinical personnel^b^
Hospital 1	Coast	4	3	14.8	8.8
Hospital 2	Central	5	8	13.9	9.2
Hospital 3	Central	4	2	13.9	9.2
Hospital 4	Western	5	5	10.0	6.3

^a^At time of the study

^b^Per 10,000 population, county-level estimates [27]

At all facilities, patient viral load samples were collected at the facility’s internal laboratory. Samples were then shipped to the facility’s designated central laboratory for processing. Each hospital’s PMTCT program was supported by the HITSystem 2.0, a web-based intervention that tracks clinical engagements during PMTCT and Early Infant Diagnosis (EID), sends providers alerts for missed or late services, and sends clients automated text messages reinforcing ART adherence and timely appointment attendance. [28] This version of the HITSystem did not have mechanisms to track or reinforce VL testing; therefore, engagement of patients in VL testing approximated the local standard of care. All pregnant women living with HIV who presented for their routine PMTCT care at the study hospital were eligible to utilize the HITSystem as part of the hospital’s standard PMTCT services. Antenatal care department (ANC) patients (n = 492) between May 2016 and March 2018 were screened for enrollment in the study.

### Data collection

PMTCT client data collected by routine entry in the HITSystem 2.0 were supplemented with data captured through retrospective review of paper-based facility records for VL testing history. Collected data included dates of ART initiation, ANC enrollment, last menstrual period (LMP), estimated date of delivery (EDD), VL sample collection, return of VL results, and clinical action in response to an unsuppressed VL result (>1000 copies/ml, per Kenyan guidelines [26]). For those without a documented infant date of birth (DOB), antenatal and postpartum testing periods were delineated using EDD. Patients who initiated ART in ANC or during the 6 months prior to LMP were defined as newly initiated. Forty-eight participants were excluded due to death or transfer, 16 were excluded for lack of both a documented LMP date and DOB, and 4 were excluded for illogically documented ANC enrollment dates. In total, 424 participants were included in analyses.

### Ethical considerations

This study was a retrospective chart review. All patient records/information was anonymized and de-identified prior to analysis. As such, retrospective review study participants were not required to provide informed consent for their clinical records to be used in this study. The Institutional Review Boards at the University of Kansas Medical Center and Kenya Medical Research Institute approved this study.”

### Analysis

VL tests occurring between the LMP date and 6 months postpartum were included in analysis, and each patient’s first VL test within this period was designated as the baseline. We assessed the proportion receiving guideline-adherent VL testing at baseline and repeat intervals. For patients newly initiated on ART, guideline adherent VL baseline testing was defined as (1) ART initiation by 14 weeks gestation and (2) baseline VL testing within 6 months of ART initiation. For patients who were previously initiated on ART, guideline-adherence baseline testing was defined as (1) occurring after LMP or at confirmation of pregnancy and (2) within the targeted timeframe of early antenatal engagement (≤14 weeks gestation). If baseline VL tests results were suppressed, repeat VL tests were indicated within 6 months of the date the baseline sample was obtained. Among those with unsuppressed baseline or repeat VL results, guidelines recommended a repeat test within 3 months. All VL samples (baseline and/or repeat) collected ≤30 days after recommended schedule were designated as guideline-adherent.

Descriptive statistics were used to report demographic characteristics of study participants, uptake of baseline and repeat VL tests, and engagement of participants with test result notification and clinical action. Chi-square tests were performed to compare proportions of patients with guideline adherent baseline and repeat VL testing among health care facilities and proportions of the previously versus newly ART-initiated patients.

## Results

Patient enrollment by year and study site are described in Table 2, as well as timing of ART initiation, maternal age, and gestational age of patients at ANC enrollment.

**Table 2:** Enrollment population characteristics.

Characteristic	No. (%)
Year of ANC enrollment	
2016	54 (12.7)
2017	342 (80.7)
2018	28 (6.6)
Facility	
#1, Coastal Kenya	65 (15.3)
#2, Central Kenya	182 (42.9)
#3, Central Kenya	67 (15.8)
#4, Western Kenya	110 (25.9)
Diagnosis and ART status	
Previously initiated on ART	250 (59.0)
Newly initiated on ART	170 (40.1)
Missing	4 (0.9)
Gestational age at delivery	
Preterm (<37 weeks)	74 (17.5%)
Early term (37 - <39 weeks_	63 (14.9%)
Full term (39 - <41 weeks)	117 (27.6%)
Late term (41 - <42 weeks)	30 (7.1%)
Postterm (≥42 weeks)	26 (6.1%)
Unknown	114 (26.9%)
	**Median (IQR)**
Maternal age at enrollment (yr)	31.0 (26, 35)
Gestational age at enrollment (wk)	23.1 (17.3, 28.9)
Time on ART at enrollment (mo)	20.4 (0, 44.5)

### Receipt of any viral load testing

Among the 424 enrolled PMTCT patients who were included in analyses, 119 women (28%) did not receive any documented VL test by 6 months postpartum. Among the 305 women (72%) who received a baseline VL test, 213 (70%) received it antenatally at a mean gestational age of 19.4 weeks and 92 (30%) received it postpartum at a mean of 12.5 weeks post-delivery. Baseline VL tests were followed by a repeat VL test within 6 months of delivery for 79 (19%) women. Twenty-six (33%) repeat VL tests were administered in the antenatal stage, 1 (1%) was administered at delivery, and the remainder were administered postpartum. Only 8% of those newly initiated on treatment received a baseline and repeat test, and only 2% of those previously initiated on treatment received a baseline and two repeat tests by 6 months postpartum (Fig 1). Those who were previously initiated on treatment were significantly more likely than those newly initiated to receive a baseline and repeat test (26.0% v. 8.2%, χ^2^ = 20.91, p < .0001). There was also a significant difference between sites for receipt of any VL testing by 6 months postpartum, with proportions ranging from 53.7% to 86.2%, χ^2^ = 20.05, p < .0001, as well as receipt of both baseline and repeat testing by 6 months postpartum, ranging from 9.9% to 29.1%, χ^2^ = 18.18, p = < .0001.

### Guideline adherent VL testing

**Fig 2:**
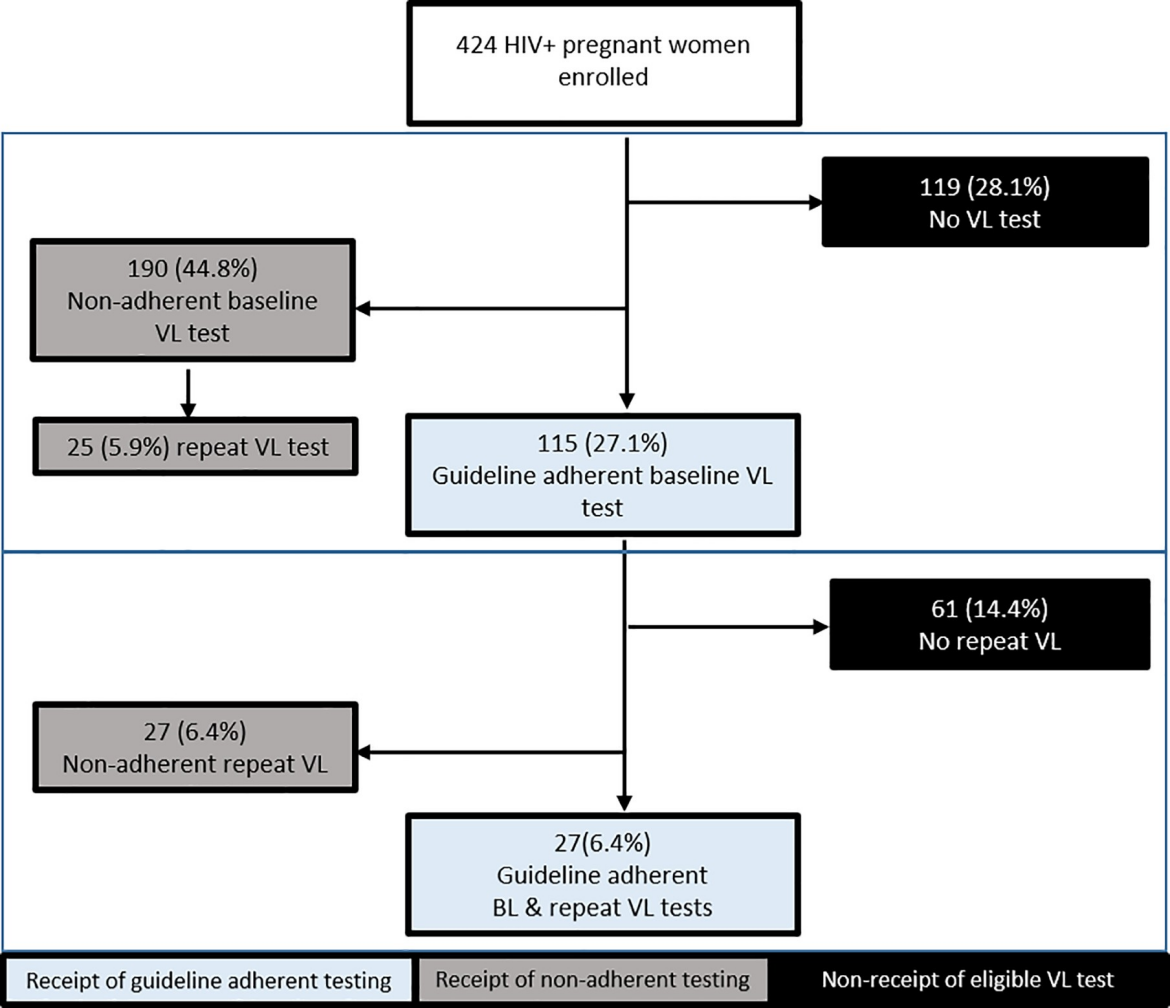
Adherence to guideline-recommended baseline and repeat viral load testing sequence, through 6 months postpartum. HIV-positive women enrolled in antenatal care clinics of four Kenyan hospitals, including patients previously initiated or newly initiated on antiretroviral therapy, were tracked retrospectively to assess guideline adherence of the viral load testing.

One hundred fifteen clients (27%) received a baseline VL test adherent to Kenya’s 2016 guidelines per their personal ART history (Fig 2). A significantly greater proportion of women who were previously initiated on ART received guideline adherent baseline VL testing, compared to women who were newly initiated on ART (34.0% v. 17.7%, χ^2^ = 13.61, p < .0001). Across the four study sites, the proportions of patients who received guideline-adherent baseline VL tests varied significantly, ranging from 14.9% to 40.0% (χ^2^ = 11.27, p = .01).

Twenty seven of the patients with guideline-adherent baseline tests subsequently received guideline-adherent repeat VL tests, while 27 others received delayed repeat testing (Fig 2). Thus, 6% of all enrolled women (27/424) received a baseline test and a repeat test that both adhered to the timing recommended in Kenya’s guidelines (Fig 2). There was no significant difference between the proportion of previously ART-initiated patients who received a guideline-adherent baseline and repeat test sequence, compared to newly initiated patients (8.0% vs 5.3%, χ^2^ = 1.11, p = .293). Receipt of the guideline-adherent baseline and repeat test sequence did not vary significantly across study sites, ranging from 4.8% to 10.9% of patients (χ^2^ = 3.87, p = .275).

### VL testing and clinical action during PMTCT

Of the 305 baseline test samples submitted to centralized laboratories, 294 (96%) VL results were reported back to the clinic. The mean turnaround time for VL results was 2.3 weeks. Patient notification was documented for only 106 (36%) of the baseline test results returned to the clinic. Thirty-eight (13%) baseline test results indicated viremia above the threshold for unsuppressed VL (≥1000 copies/ml); with no significant differences among newly ART-initiated and previously ART-initiated patients (13.8% vs 12.6%, χ^2^ = 0.08, p = 0.774). Of the 38 patients with unsuppressed VL results, 11 (29%) were followed up with documented clinical action: 9 patients were given enhanced adherence counseling and 2 others were prescribed a change in ART regimen. Additionally, 7 (18%) patients with unsuppressed baseline VL received repeat testing in accord with the guideline-recommended accelerated 3-month turnaround.

Of the 79 repeat VL test samples submitted to the centralized laboratory, 78 (99%) VL results were returned to the clinic (mean turnaround time of 2.0 weeks), and patient notification was documented for 38 (49%) patients. Fourteen (18%) of the repeat test results were unsuppressed: 2 (14%) were followed up with documented clinical action (enhanced adherence counseling) and an additional 2 (14%) had a second documented repeat test within 3 months in accordance with the guidelines.

## Discussion

Very limited prior data on VL testing uptake in Sub-Saharan African PMTCT programs suggest that only 35–50% of pregnant women on lifelong ART regimens receive any VL testing. [9, 10, 22] We are aware of no prior reports tracking the uptake of a consecutive baseline and repeat VL test sequence that fulfills PMTCT guidelines. Our data show that more than 70% of women in these four Kenyan facilities received a baseline VL test sometime during PMTCT, superior to the uptake levels reported previously. [9, 10, 22] However, only 6% of women received a baseline and repeat testing sequence adherent to the schedule in Kenya’s guidelines. Though, significant variations were observed in the uptake of guideline-adherent testing across study sites and between the newly ART-initiated and previously initiated patient populations, no site achieved more than 11% guideline adherent testing sequence for newly or previously diagnosed patients. Our results are similar to a Kenyan study that found poor uptake of repeat VL testing among non-PMTCT patients. [29] Together, this suggests a considerable need to improve the rigor and consistency of VL monitoring throughout PMTCT services–and HIV care in general—at the national level, so that delays in addressing treatment failure can be minimized.

A well-functioning clinic-laboratory interface is critical to ensure HIV diagnostic tests lead to quality, actionable results for patients. [30, 31] VL monitoring is only useful to the extent that results are returned from the laboratory, patients are notified, and any unsuppressed VL results trigger clinical intervention (e.g. adherence counseling, regimen switch). Utilization of VL results for adults living with HIV has been reported from several Sub-Saharan African settings, where ART regimen changes were documented for 14% to 56% of patients determined to have virologic failure [9, 32–35] Similarly, our data show an important deficit in test utility in Kenyan PMTCT programs. Among PMTCT clients with VL results, 13%-18% were unsuppressed at baseline and repeat testing; and among these women, less than a third received any clinical action to address their high VL. A key driver of this gap in clinical action is the low rate of documented patient notification; specifically, fewer than half of patients with unsuppressed baseline VL were notified of their test results.

Science relating to PMTCT is a rapidly evolving field of study, prompting frequent guideline changes to reflect most up-to-date evidence. [36] This study and others [37–40] suggest that, historically, the implementation of innovations into routine health settings is slow in Kenya and other sub-Saharan African countries, despite guidelines reflecting the newest evidence. The observed gap between evidence-based PMTCT guidelines and facility-level implementation is likely a result of inadequate adaptation of interventions to suit local realities–including provider knowledge and experience, health system resources and infrastructure, and patient preferences—of service provision in these areas. [41] Current dissemination efforts for new clinical guidelines are rarely prioritized at the health facility level to reach the majority of providers. The application of Implementation Science research—a field of science studying methods to promote the adoption of best practices into routine health care–may help address barriers in the adoption of PMTCT interventions, if key stakeholders including patients, clinicians, researchers, and policy- are adequately engaged. [41]

Several emerging adaptations to traditional viral load testing processes, which may help address gaps in PMTCT VL monitoring. eHealth interventions that support clinical decision making and patient outreach have proven successful at improving completion of guideline-adherent care and increasing patient notification for EID results, [42] and may be similarly effective to improve VL uptake and result notification. Point-of-care diagnostics—which allow samples to be processed at the hospital in a matter of hours and are becoming more widely utilized for VL testing–may improve result notification and provision of timely clinical action by negating the need for patient recall to the hospital and reducing loss to follow up between sample collection and result notification. Lastly, implementing differentiated care models—such as viremia clinics that allow providers to see a fewer number of patients and provide comprehensive and individualized care to patients with unsuppressed viral loads–may help further close the gap between unsuppressed viral load and clinical action. [43, 44]

Limitations of the study include the fact that all data come from patients who enrolled programmatically in the HITSystem. Although this version of the intervention provided no direct support for VL testing adherence, it surpasses the local standard of care and may bias the results toward greater ART and appointment adherence. If so, the true proportions of women receiving VL tests and VL-driven clinical action are likely even lower. Also, the population enrolled in this study, at four hospitals, is a small sample of PMTCT clients and facilities nationwide. Despite that constraint, the chosen study sites represent diverse settings from the standpoint of geographic region and facility size, so documented engagement in services there are likely to reflect the situation in many Kenyan localities. In addition, the timing and duration of antenatal PMTCT engagement varied at the individual level, thus patients who received late baseline testing (after delivery) may not have been eligible for retesting by 6 months postpartum. Furthermore, this study is limited to retrospective review of facility records, and thus, does not include exploration of site-specific factors that may have influenced uptake and compliance with guidelines. Nonetheless, these data accurately describe the proportion of women receiving guideline adherent antenatal enrollment and VL monitoring between the targeted intervention window of pregnancy through 6 months postpartum.

Unique contributions of this study include that it followed patients longitudinally and thus captured maternal VL testing uptake both antenatally and postpartum, including baseline and repeat testing. By collecting the documented dates of VL testing and VL-driven clinical action–in conjunction with dates of ANC enrollment, ART initiation, and delivery–we were able to measure for each patient the adherence to Kenya’s national PMTCT guidelines. An important feature of this study was the analysis of clinical actions taken in response to high VL test results, providing insight into the fulfillment of VL testing’s intended purposes.

The investment in VL monitoring during pregnancy and postpartum can avert perinatal HIV transmission and protect women’s health. Data collected from PMTCT programs at these sites suggest the need for enhanced communication channels between providers, central laboratories, and patients in order to reinforce guideline-adherent test orders, patient notification, and rapid clinical action. Meeting these goals is critical to realizing a maximum return on current investment and optimizing PMTCT outcomes.

## Supporting information

S1 Dataset (XLS)
